# Supplementary material for: MetaRibo-Seq measures translation in microbiomes
Source: Nat Commun. 2020 Jun 29;11:3268. doi: 10.1038/s41467-020-17081-z (PMC7324362; doi:10.1038/s41467-020-17081-z)
Supplement: Supplementary file 10 — Supplementary Data 7 [file 41467_2020_17081_MOESM10_ESM.zip › File2/Confidence_High_Taxonomy/212620_out.krona.html]

Javascript must be enabled to view this page.

members
magnitude
magnitudeUnassigned
count
unassigned
taxon
rank

212620\_out

5

superkingdom
5
2

phylum
976
5

200643
5
class

171549
5
order

815
5
family

genus
5
816


SRS077849\_contig\_number\_21780SRS144603\_contig\_number\_12425SRS146812\_contig\_number\_45527SRS146813\_contig\_number\_1118SRS148196\_contig\_number\_28837
species
5
46506
